# CircFNDC3B sequestrates miR‐937‐5p to derepress TIMP3 and inhibit colorectal cancer progression

**DOI:** 10.1002/1878-0261.12796

**Published:** 2020-09-19

**Authors:** Wei Zeng, Yi Liu, Wen‐Ting Li, Yi Li, Jin‐Feng Zhu

**Affiliations:** ^1^ Department of Hematology and Oncology Shenzhen University General Hospital Shenzhen China; ^2^ Shenzhen University International Cancer Center China; ^3^ Department of Cardiothoracic Surgery Shenzhen University General Hospital Shenzhen China; ^4^ Department of Pathology Shenzhen University General Hospital Shenzhen China; ^5^ Department of General Surgery Shenzhen University General Hospital Shenzhen China

**Keywords:** angiogenesis, circFNDC3B, colorectal cancer, liver metastasis, miR‐937‐5p, TIMP3

## Abstract

Circular RNA (circRNA) are single‐stranded RNA with covalently closed 3′ and 5′ ends, with many recognized to be involved in human diseases as gene regulators, typically by interacting with other RNA. CircFNDC3B is a circRNA formed by back‐splicing of exons 5 and 6 of the FNDC3B gene. CircFNDC3B was recently implicated in renal carcinoma, gastric and bladder cancer. However, the expression levels of circFNDC3B and its role in colorectal cancer (CRC) remain unclear. Expression of circFNDC3B and TIMP3 levels in CRC tissues and cell lines were found to be low, whereas microRNA (miR)‐937‐5p expression was high in CRC. MicroRNA‐937‐5p downregulated TIMP3, thereby promoting tumor cell proliferation, invasion, migration and angiogenesis. Moreover, CircFNDC3B was shown to bind to miR‐937‐5p. CircFNDC3B and circFNDC3B‐enriched exosomes inhibited tumorigenic, metastatic and angiogenic properties of CRC, and miR‐937‐5p overexpression or TIMP3 knockdown could reverse these effects. *In vivo* CRC tumor growth, angiogenesis and liver metastasis were suppressed by circFNDC3B overexpression, circFNDC3B‐enriched exosomes or miR‐937‐5p knockdown. In conclusion, our work reports a tumor‐suppressing role for the circFNDC3B–miR‐97‐5p–TIMP3 pathway and suggests that circFNDC3B‐enriched exosomes can inhibit angiogenesis and CRC progression.

## Graphical abstract

CircFNDC3B induced TIMP3 to suppress angiogenesis to inhibit CRC cell proliferation, migration and invasion by negatively targeting miR‐937‐5p. Also, circFNDC3B exosomes could repress CRC angiogenesis by decreasing VEGFR expression.

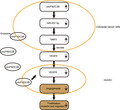

**Abbreviations**
ANOVA: analysis of variance
CCK‐8: Cell Counting Kit‐8
circRNA: circular RNA
CM: conditioned medium
CRC: colorectal cancer
DMEM: Dulbecco’s modified Eagle’s medium
EM: extracellular matrix
EMT: epithelial‐mesenchymal transition
FISH: fluorescence innonbreakingspacesitu hybridization
GO: gene ontology
HUVEC: human umbilical vein endothelial cell
IHC: immunohistochemistry
KEGG: Kyoto Encyclopedia of Genes and Genomes
MiR, microRNA
MMPs: matrix metalloproteinases
NTA: nanoparticle tracking analyzing
RIP: RNA immunoprecipitation
sh‐, short hairpin‐
SD: standard deviation
STRING: Search Tool for the Retrieval of Interacting Genes/Proteins
TEM: transmission electron microscope
TIMP3: tissue inhibitor of metalloproteinase 3

## 1 Introduction

Colorectal cancer (CRC), also called bowel cancer, rectal cancer or colon carcinoma, is the third most commonly diagnosed cancer worldwide [1] and accounts for almost 900 000 deaths per year as the second leading cause of cancer‐related death [2]. CRC has 5‐year survival rate if detected early and properly treated. However, since CRC often develops no obvious symptoms during early stages, many patients are diagnosed at advanced stages and already have metastasis [3]. Metastases in CRC patients, especially liver metastasis, are frequently associated with a very poor prognosis and higher risk of death [4, 5]. Treating the patients with CRC, particularly those who have a distant metastasis, presents a key challenge and most of the currently available treatment options are eventually palliative [6, 7]. Therefore, it is imperative to better understand the underlying molecular mechanisms of CRC progression and metastasis development and create more efficient therapeutic approaches to cure this malignant disease.

Circular RNA (circRNA) are a class of single‐stranded RNA that are covalently closed by linking the 3′ and 5′ ends, and many of them have been recognized to be involved in various human diseases as gene regulators [8]. CircFNDC3B is a circRNA that is formed by back‐splicing exons 5 and 6 in the FNDC3B gene and has recently been implicated in gastric cancer [9], cardiovascular disease [10], bladder cancer [11] and renal carcinoma [12] by modulation of specific signaling pathways. However, the expression level of circFNDC3B and its underlying molecular mechanisms in CRC have never been reported. Exosomes are a type of small extracellular vesicle that can selectively transport proteins, DNA, RNA and lipids to target cells and have emerged as an important mediator in intracellular communication during last decade [13, 14]. Notably, it has been demonstrated in previous reports that circRNA are enriched in human exosomes [15, 16] and have profound impacts on cell proliferation [17], tumor metastasis [18, 19] and oncogenic drivers [20]. However, little is known about the biological function of circFNDC3B in exosomes. In our present study, we demonstrate for the first time a direct binding between circFNDC3B with microRNA‐937‐5p (miR‐937‐5p). MicroRNA is a type of non‐coding RNA molecule of 21–25 nucleotides in length and is considered to be responsible for regulating their target genes that are involved in significant pathways. Aberrant expression of miR‐937‐5p has been observed in malignant pleural mesothelioma [21], nasopharyngeal carcinoma [22], rectal cancer [23] and gestational diabetes [24]. However, the regulatory role of miR‐937‐5p in CRC has not been studied in depth, nor has the regulatory interaction between circFNDC3B and miR‐937‐5p been investigated or reported until now.

Tissue inhibitor of metalloproteinase 3 (TIMP3) is one of the four members of the TIMP family which act as major regulators of the matrix metalloproteinases (MMPs). Dysregulated expression of TIMP3 was observed in clear cell renal cell carcinoma [25], breast cancer [26] and colon cancer [27]. Overexpression of TIMP3 was indicated to inhibit cell proliferation and migration in colon cancer [27, 28], melanoma [29, 30] and other human cancers [31]. On the other hand, TIMP3 was proved to function as a tumor‐suppressor by blocking the binding between VEGF and VEGFR [32] and modulating extracellular matrix (EM) production, an important player in angiogenesis [33]. Here we demonstrate for the first time that TIMP3 is a potential target of miR‐937‐5p based on our bioinformatics analysis and provide the first evidence for the specific binding sites between these two molecules in CRC.

In the present study, we conducted both *in vitro* and *in vivo* studies and found that circFNDC3B and TIMP3 expression levels were downregulated, whereas miR‐937‐5p was upregulated in CRC tissues and cells. We experimentally reasoned that circFNDC3B negatively and directly regulated miR‐937‐5p to induce the expression level of the tumor‐suppressor TIMP3, which inhibiting the metastasis, invasion and angiogenesis of CRC. Thus, circFNDC3B performed a key role in CRC tumorigenesis and metastasis. Furthermore, we also found that tumor growth and angiogenesis were effectively inhibited by exosomes collected from circFNDC3B overexpressing CRC cell lines. By highlighting the regulatory interactions of circFNDC3B, miR‐937‐5p and TIMP3, our research work provided insights into CRC pathophysiological mechanism and could be helpful for future development of innovative therapeutics.

## 2 Materials and methods

### 2.1 Bioinformatic analysis of circRNA expression profiles

Datasets containing normalized expression profiles of exosome circRNA based on RNA‐seq data were downloaded from exoRBase (http://www.exorbase.org/). Thirty‐two plasma samples were obtained from normal individuals and 12 from CRC patients. The expression profiles were filtered prior to the difference analysis by using the criterion that circRNA expression was detectable from at least 10 samples in each group. Difference analysis was performed using the R/bioconductor software limma package. Gene ontology (GO) enrichment analysis was conducted on the 387 circRNA which were differentially expressed in two groups and the metastasis‐associated genes were identified.

TIMP3 regulation pathways were predicted by searching the Kyoto Encyclopedia of Genes and Genomes database (KEGG). The interaction between TIMP3 and VEGFA was computed and visualized using Search Tool for the Retrieval of Interacting Genes/Proteins (STRING).

### 2.2 Human tissue sample collection and ethics statement

This study and all the experimental procedures were approved by the Ethics Committee of Shenzhen University General Hospital (Shenzhen, Guangdong, China). Twenty cases of fresh CRC tumor tissues and adjacent normal tissues were collected from CRC patients who were diagnosed and treated at Shenzhen University General Hospital. The collected samples were snap‐frozen in liquid nitrogen and preserved at −80 °C. Written informed consent was obtained from participants and detailed clinical data of CRC patients in this study are provided in Table 1. All experiments were conducted in strict accordance with the Declaration of Helsinki.

**Table 1:** Clinicopathological characteristics in patients with CRC.

Characteristics	Cases (*n*)
Age (years)	
< 60 years	6
≥ 60 years	14
Sex	
Male	10
Female	10
Histological type	
Adenocarcinoma	11
Mucinous adenocarcinoma	9
TNM stage	
I–II	8
III–IV	12
Tumor size (cm)	
< 1.5	5
≥ 1.5	15
Liver metastasis	
Yes	13
No	7

### 2.3 Cell culture

Human CRC cell lines LoVo, SW480, SW620 and HCT116, normal colonic epithelial cell line FHC, human umbilical vein endothelial cells (HUVEC) and 293T cells were purchased from the American Type Culture Collection (Manassas, VA, USA). All the cells were cultured in Dulbecco’s modified Eagle’s medium (DMEM, Gibco, Carlsbad, CA, USA), which was supplemented with 10% FBS (Gibco), 1% penicillin (Invitrogen, Carlsbad, CA, USA) and streptomycin (Invitrogen). Medium was changed every second day. For exosome isolation experiment, cells were transferred into DMEM containing 10% exosome‐depleted FBS (Gibco). Cells were incubated at 37 °C in a humidified chamber that contained 5% CO_2_.

### 2.4 Exosome isolation

Exosomes were isolated from the cell culture supernatant using ultracentrifugation method as previously described with minor modifications [34]. Briefly, cells were planted in a 10‐cm culture dish (Sigma, St. Louis, MO, USA) and transferred to new medium containing 10% exosome‐depleted FBS at 50% confluence. The cell cultures were further incubated for 48 h and cellular debris was removed by centrifugation at 2000 ***g*** for 30 min. Subsequently, the supernatant was subjected to ultracentrifugation at 100 000 ***g*** for 2 h. The pellets were dissolved in PBS and then sub‐fractioned by centrifugation at 17 000 ***g*** for 30 min and 100 000 ***g*** for 30 min. The exosome pellets were recovered in 500 µL TRIzol for Q‐PCR experiment, 200 µL RIPA buffer for western blot analysis or PBS for nanoparticle tracking analyzing (NTA) and transmission electron microscope (TEM) analysis.

### 2.5 Exosome characterization

Morphology of isolated exosomes was examined under TEM according to the method previously described [35]. Exosome samples were prepared by using Exosome‐TEM‐easy Kit (101Bio; Palo Alto, San Francisco, CA, USA) based on the manufacturer’s guidelines. Briefly, exosomes were adsorbed on the formvar‐carbon‐coated mesh 400 grid for 10 min and stained in EM solution in the dark for 10 min. The samples were washed, transferred to a Whatman Grade 1 Filter Paper (Sigma) and air‐dried in the dark. TEM images were taken using a Zeiss EM109 TEM (Carl Zeiss, Oberkochen, Germany) at 80 kV accelerating voltage.

The exosomes purified above were diluted 1 : 100 and NTA was performed to validate the size and concentration of exosomes using a NanoSight NS500 instrument (Malvern Instruments Ltd, Malvern, UK) as previously described [36].

### 2.6 RNase R digestion and circFNDC3B half‐life determination

CircFNDC3B and its linear counterpart FNDC3B mRNA were digested by the RNase R. A 2‐µg aliquot of total RNA was incubated for 30 min at 37 °C with and without 0.5 μL 10 × RNase R Reaction Buffer and 0.2 μL RNase R. The relative expression levels of circFNDC3B and FNDC3B mRNA were then measured by Q‐PCR. Stability of circFNDC3B and linear FNDC3B was quantified by calculating their half‐lives. Cells were treated with 2 µg·mL^−1^ actinomycin D (Sigma) to block the gene transcription and total RNA was harvested at the indicated time points (0, 4, 8, 12, 24 h). Relative expression levels of both transcripts were detected by Q‐PCR and their half‐lives were determined.

### 2.7 Cellular detection of circFNDC3B and miR‐937‐5p by fluorescence *in situ* hybridization

To visualize the localization of circFNDC3B and miR‐937‐5p, LoVo and SW480 cells that were seeded on coverslips were grown to 80% confluence and fixed in 4% paraformaldehyde for 20 min. The cells were permeabilized with 0.1% Triton X‐100 in PBS for 10 min and subjected to fluorescence *in situ* hybridization (FISH) using circFNDC3B or miR‐937‐5p‐specific probes. RNA oligonucleotide probes were generated by Shanghai Invitrogen Biotechnology (Shanghai, China). LoVo and SW480 cells were pre‐hybridized in 1 × PBS/0.5% Triton X‐100 and hybridized with the biotinylated RNA probes in hybridization buffer (40% formamide, 10% dextran sulfate, 4 × SSC, 10 mm DDT and 1 mg·mL^−1^ yeast transfer RNA) at 37 °C overnight. The FISH signals of circFNDC3B or miR‐937‐5p probes were detected using the Alexa Fluor 488 Signal‐Amplification Kit, and images were acquired using a Nikon confocal microscope (Eclipse TE2000U; Nikon, Tokyo, Japan).

### 2.8 Cell transfection

CircFNDC3B, short hairpin (sh)‐circFNDC3B, miR‐937‐5p mimics, miR‐937‐5p inhibitor, sh‐TIMP3 or their negative controls were synthesized by Sangon Biotech (Shanghai, China) and inserted into a lentivirus pLCDH vector for stable expression or knockdown. Next, lentivirus was amplified and purified, and viral titer was determined by plaque assay (10^8^ PFU·mL^−1^). LoVo and SW480 cells were seeded on a 96‐well plate at a cell density of 1.5 × 10^4^ cells·well^−1^ and grown to 60% confluence before transduction. Lentiviral particles were mixed with polybrene (10 µg·mL^−1^) and added into the cells at multiplicity of Infection (MOI) of 10. Cells were incubated at standard cell culture condition for another 48 h and ready for further analysis.

### 2.9 Luciferase reporter assay

Putative binding sites between circFNDC3B and miR‐937‐5p were predicted by sequence alignment, and binding sites between miR‐937‐5p and TIMP3 were predicted using the web servers miRanda and TargetScan, respectively. Mutations of the predicted binding sites were generated using a QuickChange Site‐directed Mutagenesis Kit (StrataGene, Shanghai, China). 3’‐UTR of wild‐type TIMP3 (TIMP3‐WT), circFNDC3B (circFNDC3B‐WT) and their mutants (circFNDC3B‐MUT and TIMP3‐MUT) were sub‐cloned into psiCHECK2 dual‐luciferase vector (Promega, Madison, WI, USA). On a 12‐well culture plate, 293T cells were seeded and grown to 50% confluence. Cells were co‐transfected with the luciferase reporter vectors above and miR‐937‐5p mimics or negative control (mimics NC) using Lipofectamine 2000 (Invitrogen). At 48 h after transfection, cells were harvested and lysed. Luciferase activities were measured using Dual‐Luciferase Reporter Assay System (Promega) according to the manufacturer’s protocols, and relative Renilla luciferase activities were normalized to firefly luciferase expression.

### 2.10 RNA immunoprecipitation

The direct binding between circFNDC3B and miR‐937‐5p was further evaluated by RNA immunoprecipitation (RIP) assay. RIP was carried out using EZ‐Magna RIP RNA‐Binding Protein Immunoprecipitation Kit (Millipore, Billerica, MA, USA) according to the manufacturer’s protocol. Briefly, 293T cells were transfected with miR‐937‐5p mimics or mimics NC at 60% confluence and harvested after 48 h. The cells were lysed in RIP lysis buffer supplemented with protease inhibitor cocktail (Roche, Basel, Switzerland) and ribonuclease inhibitor (Sigma) and incubated with magnetic beads conjugated to human anti‐Argonaute 2 antibody (1 : 50; Millipore) or the nonspecific anti‐IgG antibody (Millipore) as negative control at 4 °C overnight. After proteinase K treatment, the immunoprecipitated RNA was eluted and subjected to Q‐PCR analysis to determine circFNDC3B enrichment.

### 2.11 CCK‐8 assay

LoVo and SW480 cells were planted in a 96‐well plate at a cell density of 2000 cells·well^−1^ and transfected with lentiviruses after indicated treatment. Cell proliferation was measured using Cell Counting Kit‐8 (CCK‐8; Dojindo, Kumamoto, Japan) according to the user’s instructions. Briefly, 10 µL of CCK‐8 assay reagent was added into every well and incubated at 37 °C for 2 h. The optical density at 450 nm was detected with a microplate reader (Tecan Infinite 200 Pro, Männedorf, Switzerland).

### 2.12 Colony formation assay

LoVo and SW480 cells were infected with lentiviruses after indicated treatment and maintained at 37 °C. Cells were diluted and re‐planted in a six‐well plate at a density of 100 cells·well^−1^. After incubation for 7 days, colonies were fixed with 3.7% methanol and stained with 0.5% crystal violet at room temperature for 2 h. The number of colonies was counted under a microscope (Carl Zeiss).

### 2.13 Wound‐healing assay

LoVo and SW480 cells were seeded in six‐well plates after indicated treatment. Forty‐eight hours after transfection, the monolayer was gently scratched using a new 10‐µL pipette tip. For the wound‐healing experiment in HUVEC, conditioned medium (CM) was collected from LoVo and SW480 cells that were transfected with the viruses above and incubated with HUVEC. Detached cells were carefully washed away with PBS, and the remaining cells were replenished with fresh serum‐free medium. The wound‐healing processes were recorded by the microscope (Carl Zeiss).

### 2.14 Invasion assay

Transwell assays were performed to study the cell invasion capacity using Transwell permeable supports (8 μm; Corning, Corning, NY, USA). LoVo and SW480 cells were infected with lentiviruses after indicated treatment. The cells were then harvested and diluted in serum‐free DMEM. An aliquot of 5 × 10^4^ cells was seeded in the upper chamber which was pre‐coated with Matrigel (BD Biosciences, Franklin Lakes, NJ, USA), whereas fresh DMEM containing 10% FBS was added to the bottom chamber. After further incubation for 48 h, the invaded cells were fixed in 4% paraformaldehyde, stained with 1% crystal violet and counted under the microscope (Carl Zeiss).

### 2.15 ELISA

LoVo and SW480 cells were infected with lentiviruses after indicated treatment and incubated at 37 °C for 48 h. VEGFA expression levels were quantified using a human VEGFA ELISA Kit (Abcam, Cambridge, UK, ab119566) essentially according to the manufacturer’s protocol. The absorbance of both samples and standards at 450 nm was recorded on the microplate reader.

### 2.16 *In vitro* HUVEC tube formation assay

HUVEC 5 × 10^4^ were seeded on a 24‐well culture plate which was pre‐coated with ice‐cold Matrigel (BD Biosciences). CM was collected from LoVo and SW480 cells that were transfected with the viruses above and incubated with HUVEC for 24 h. The angiogenesis was imaged with the microscope (Carl Zeiss) and evaluated by counting the number of junctions under the imagej software (NIH, Bethesda, MD, USA).

### 2.17 *In vivo* CRC tumor generation and exosome injection *via* tail vein

All the animal experiment procedures were approved by the Animal Ethics Committee of Shenzhen University General Hospital (Shenzhen, Guangdong, China). LoVo cells were infected with lentiviruses carrying circFNDC3B, miR‐937‐5p inhibitor or their negative controls and harvested 48 h after transfection. Six‐week‐old male BALB/C nude mice (16–18 g) were obtained from Shanghai SLAC Laboratory Animal Center (Shanghai, China) and randomized into six groups (*n* = 6 per group). Mice were subcutaneously injected with 100 µL of 10^7^ LoVo cells in serum‐free DMEM supplemented with 50% Matrigel. For liver metastasis analysis, LoVo cells were injected into mice *via* the tail vein. In some experiments involved in exosomes, cancer cells without any treatment were subcutaneously injected and exosomes were injected *via* the tail vein. Tumor sizes as well as weights were measured. Tumor volumes were calculated using the formula: *V* = 0.5 × length × width^2^. After 32 days following injection, mice were sacrificed and tumor tissues were collected for further analyses.

### 2.18 Immunohistochemistry staining of TIMP3 and VEGFR

Tumor tissues were fixed in 10% neutral buffered formalin (Sigma) over 24 h and embedded in paraffin. Consecutive tissue sections of 5 µm were cut in a cryostat and adhered to the histological slides pre‐coated with amino‐propyl‐triethoxy‐silane (Sigma). The slides were then deparaffinized in xylene and rehydrated in graded alcohols, followed by quenching of endogenous peroxidase activity with high‐pressure heat treatment. The sections were incubated with primary antibodies against TIMP3 and VEGFR at 4 °C overnight. The slides were washed the next day and incubated with HRP‐conjugated secondary antibody at room temperature for 1 h. Nuclear was counterstained with hematoxylin (Sigma), mounted with Aquatex (Merck, Darmstadt, Germany) and photomicrographs were captured under a microscope (Carl Zeiss).

### 2.19 Western blot analysis

Cells and purified exosomes were lysed in RIPA buffer (Sigma) containing protease inhibitor cocktail (Roche) and total protein concentration was tested using bicinchoninic acid protein determination kit (Sigma). Aliquots of 25 µg of proteins were separated on SDS/PAGE gel and transferred to a poly(vinylidene difluoride) membrane. Then the membranes were blocked with 5% BSA, incubated with primary antibodies against CD163 (1 : 1000; Cell Signaling Technology, Danvers, MA, USA), TSG101 (1 : 2000; Abcam), E‐cadherin (1 : 1500; Cell Signaling Technology), N‐cadherin (1 : 1000; Cell Signaling Technology), MMP‐9 (1 : 2000; Cell Signaling Technology), MMP‐2 (1 : 2000; Cell Signaling Technology), slug (1 : 3000; Cell Signaling Technology), VEGFR (1 : 1000; Cell Signaling Technology), TIMP3 (1 : 1000; Cell Signaling Technology), GADPH (1 : 5000; Cell Signaling Technology) or β‐actin (1 : 5000; Cell Signaling Technology) overnight at 4 °C. The blots were washed, incubated with HRP‐conjugated secondary antibody (1 : 5000; Cell Signaling Technology) at room temperature for 1 h, and proteins were detected using Pierce ECL Western Blotting Substrate under an Odyssey infrared scanner (LI‐COR Biosciences Inc., Lincoln, NE, USA). Western blot bands were analyzed with NIH imagej software.

### 2.20 Total RNA extraction, Q‐PCR and Sanger sequencing

Total RNA from snap‐frozen tissue samples, cell lines or exosomes was isolated with TRIzol reagent (Takara, Dalian, China). RNA purity and concentration were measured by UV‐spectroscopy at 260 and 280 nm. RNA 1 µg was reversely transcribed using Takara PrimeScript RT Reagent Kit. Real‐time qPCR was performed with the SYBR Premix Dimer Eraser Kit (Takara) *via* the StepOnePlus Real‐Time PCR system (Applied Biosystems, Thermo Fisher Scientific, Inc., Waltham, MA, USA). Expression levels of mRNA, miRNA and circRNA were normalized to the internal control GAPDH or U6. Fold changes of relative expression were calculated using 2^−ΔΔCt^ methods. Primer sequences used for qRT‐PCR were purchased from Sangon Biotech Co., Ltd. To confirm the ‘head‐to‐tail’ back‐splicing of circFNDC3B, PCR was performed and products cloned into pGEM‐T Easy vector were subjected to Sanger sequencing.

### 2.21 Statistical analysis

All the experiments were repeated at least three times. Data were analyzed with graphpad prism 6.0 (San Diego, CA, USA) and results were expressed as mean ± standard deviation (SD). Statistical evaluation was performed using Student’s *t* test (two‐tailed) between two groups or one‐way analysis of variance (ANOVA) followed by Tukey’s *post hoc* test for multiple comparison. Spearman correlation analysis was performed to analyze the correlation between circFNDC3B, miR‐937‐5p and TIMP3 in CRC tissues. *P* values < 0.05 were considered statistically significant for all analyses: *P < 0.05; **P < 0.01; ***P < 0.001.

## 3 Results

### 3.1 Expression profiles of circRNA in CRC tissues, cells and exosomes

**Fig. 1:**
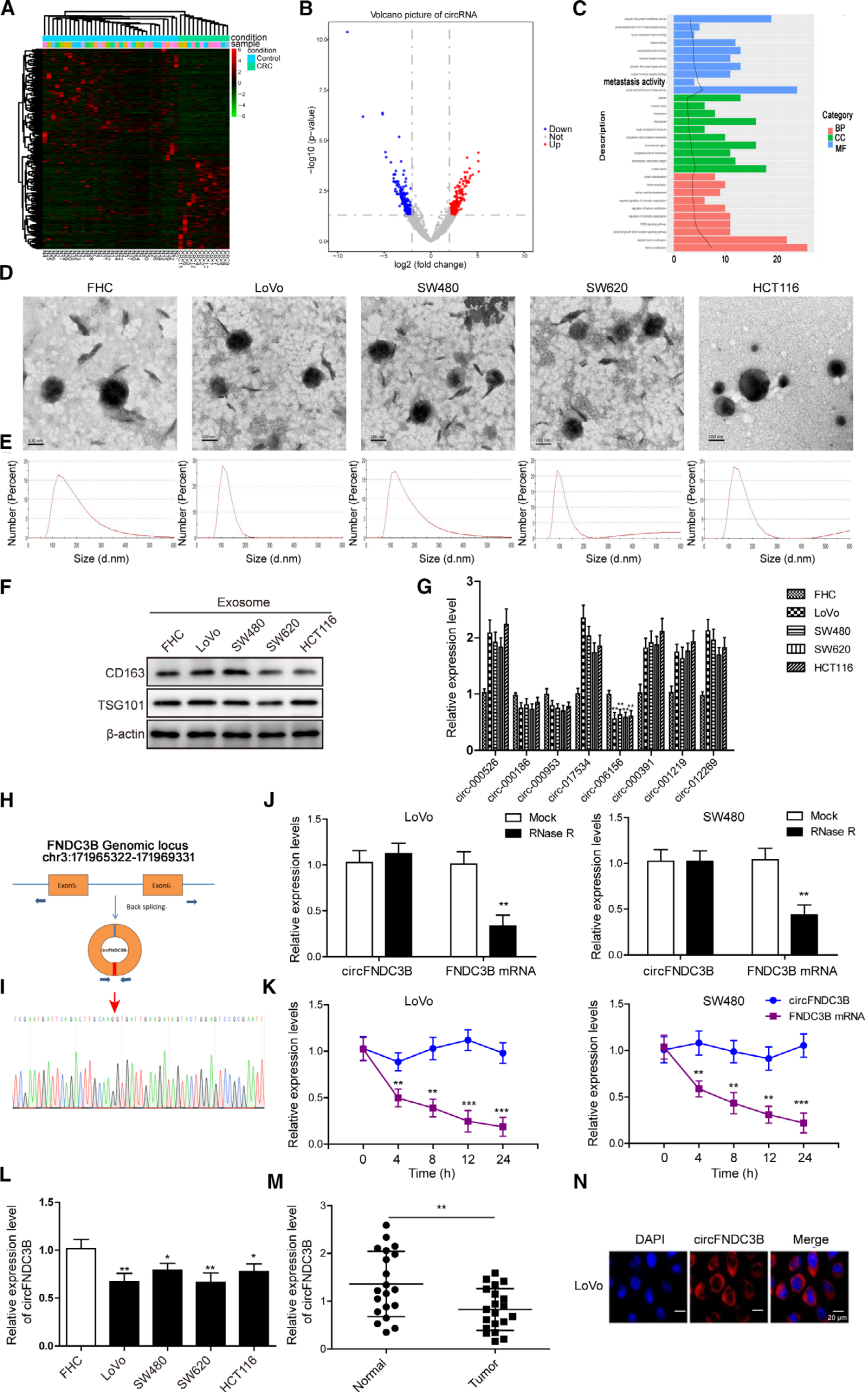
Expression profiles of circRNA in CRC tissues, cells and exosomes. (A) Heat map of the 387 differentially expressed circRNA in plasma exosomes which were isolated from CRC patients (*n* = 12) or healthy individuals (*n* = 32). (B) Differences of circRNA expression between plasma exosomes from CRC patients and healthy controls were demonstrated in volcano plot. (C) GO enrichment analysis was performed and the exosomal circRNA associated with metastasis were identified. (D) Representative TEM images of exosomes isolated from different cell culture CM showing the typical exosome morphology and size distribution. Scale bar: 100 nm. (E) Size and concentration of the exosomes were determined by NTA. (F) Western blot was performed to determine the expression of exosome protein markers CD163 and TSG101. (G) Relative expression levels of eight circRNA in exosomes isolated from CRC cells (*n* = 4) and normal colonic epithelial cells (FHC) were determined by Q‐PCR. (H) CircFNDC3B was formed by the back‐splicing of exons 5–6 in the FNDC3B gene. (I) CircFNDC3B formed by the back‐splicing of exons 5–6 in FNDC3B gene was confirmed by Sanger sequencing. (J) Cell lysate was treated with RNase R and relative expression levels of circFNDC3B as well as linear FNDC3B were examined by Q‐PCR. (K) Half‐life of circFNDC3B and linear FNDC3B was determined by measuring their relative expression levels at 0, 4, 8, 12 and 24 h. (L) Relative expression levels of circFNDC3B in the four CRC cells and FHC cells were compared. (M) CircFNDC3B was differentially expressed in tissue samples from healthy individuals and CRC patients (*n* = 20). (*N*) CircFNDC3B location in LoVo cells by FISH assay. Scale bar: 20 μm. All the experiments were repeated three times. Statistical evaluation was performed using Student’s *t* test (two‐tailed) between two groups or one‐way ANOVA followed by Tukey’s *post hoc* test for multiple comparison. Data are expressed as mean ± SD. *P < 0.05; **P < 0.01; ***P < 0.001.

To answer the question whether circRNA expression was dysregulated in CRC, we downloaded gene expression datasets from exoRBase [37] and compared the circRNA expression in plasma exosomes isolated from CRC patients (*n* = 12) and healthy individuals (*n* = 32). In all, we identified 387 circRNA that were differentially expressed between two groups, among which 176 were significantly upregulated and 211 were downregulated in CRC exosome specimens (Fig. 1A,B, Table 2). GO enrichment analysis is commonly recognized as an important tool in the functional annotation of cellular systems [38]. Next, we performed GO analysis with circRNA and found that eight of the 387 circRNA were associated with metastasis (Fig. 1C). To investigate the role of exosomal circRNA in CRC, we first isolated exosomes from cell culture CM secreted by FHC, LoVo, SW480, SW620 and HCT116 cells and observed the typical morphology of exosomes under TEM (Fig. 1D). Purified exosomes were further characterized by NTA (Fig. 1E) for size and concentration, and western blot analysis (Fig. 1F) by detecting the specific markers (CD163 and TSG101). We then measured the relative expression of the eight circRNA selected by GO enrichment analysis above, in exosomes collected from the five indicated cell lines. We observed that circ‐006156 (circFNDC3B) was obviously downregulated in all four CRC cell lines as compared with the normal colonic cell line (Fig. 1G). As shown in Fig. 1H, circFNDC3B was produced by the back‐splicing of exons 5–6 in FNDC3B gene, and the splice junction of circFNDC3B was verified by performing Sanger sequencing (Fig. 1I). Next, we compared the stability of circFNDC3B with that of linear FNDC3B. As illustrated in Fig. 1J,K, circFNDC3B was more resistant to RNase R degradation and exhibited a much longer half‐life than did linear FNDC3B. Consistent with the exosomal results, circFNDC3B expression was also significantly lower in CRC cells (Fig. 1L) as well as CRC tissue samples (Fig. 1M) than in controls. Additionally, our FISH results showed that circFNDC3B was located predominantly in cytoplasm (Fig. 1N). These results showed that numerous circRNA were aberrantly expressed in CRC patients. In particular, circFNDC3B expression was significantly decreased in CRC tissues, CRC cell lines and exosomes.

**Table 2:** Differentially expressed circRNA in plasma exosomes isolated from CRC patients and healthy individuals.

	Upregulation	Downregulation	Total	Threshold value
circRNA	176	211	387	|log_2_FC| > 2, *P* value < 0.05

### 3.2 CircFNDC3B directly target miR‐937‐5p in CRC

**Fig. 2:**
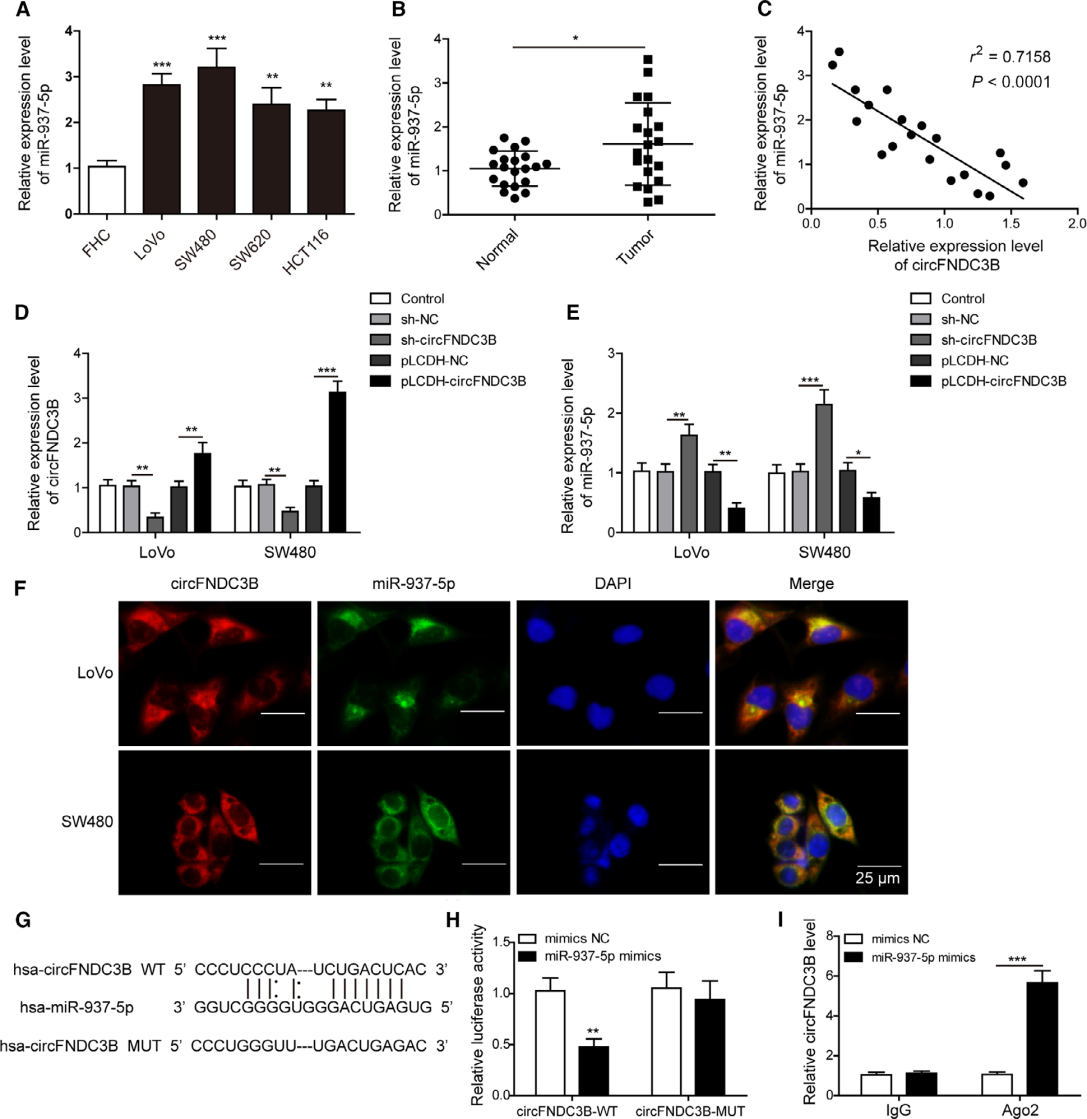
CircFNDC3B directly targeted miR‐937‐5p in CRC. (A) Q‐PCR was performed to determine the expression of miR‐937‐5p in FHC and indicated CRC cell lines. (B) Relative expression levels of miR‐937‐5p were compared in healthy individuals and CRC patients (*n* = 20). (C) Correlation analysis between the expression of circFNDC3B and miR‐937‐5p in CRC tissues. (D, E) Cells were treated with sh‐circFNDC3B, pLCDH‐circFNDC3B or their negative controls and the relative expression levels of circFNDC3B (D) as well as miR‐937‐5p (E) were determined by Q‐PCR. (F) The colocalization of circFNDC3B and miR‐937‐5p was tested by FISH experiment. Scale bar: 25 μm. (G) The putative binding between circFNDC3B and miR‐937‐5p was predicted by sequence alignment and potential binding sites are shown. (H) 293T cells were co‐transfected with miR‐937‐5p mimics or mimics NC and circFNDC3B‐WT or circFNDC3B‐MUT luciferase reporter plasmids. Cells were harvested 48 h after transfection and relative luciferase activity was recorded. (I) RIP was performed to confirm further the direct binding between circFNDC3B and miR‐937‐5p. All the experiments were repeated three times. Statistical evaluation was performed using Student’s *t* test (two‐tailed) between two groups or one‐way ANOVA followed by Tukey’s *post hoc* test for multiple comparison. Data are expressed as mean ± SD. *P < 0.05; **P < 0.01; ***P < 0.001.

We detected markedly increased expression of miR‐937‐5p in LoVo, SW480, SW620 and HCT116 cells in comparison with FHC (Fig. 2A). Consistently, tumor tissues in CRC patients also displayed higher miR‐937‐5p expression than found in healthy individuals (Fig. 2B). Moreover, the expression levels of circFNDC3B and miR‐937‐5p in CRC tissue samples exhibited a significant negative correlation (Fig. 2C). To elucidate the regulatory relationship between circFNDC3B and miR‐937‐5p, we infected LoVo and SW480 cells with lentiviruses encoding circFNDC3B or sh‐circFNDC3B for stable expression or knockdown (Fig. 2D). Remarkably, the relative expression of miR‐937‐5p was largely increased after sh‐circFNDC3B transfection, and decreased upon circFNDC3B overexpression in both cell lines (Fig. 2E). These results together confirmed our speculation that circFNDC3B regulated miR‐937‐5p, expression levels of which were negatively correlated in CRC. To identify specific interaction between circFNDC3B and miR‐937‐5p, we first conducted FISH experiment and observed the cytoplasmic colocalization of circFNDC3B and miR‐937‐5p (Fig. 2F). Next, we applied sequence alignment and predicted that miR‐937‐5p was a potential binding partner of circFNDC3B (Fig. 2G). The putative binding was tested using luciferase reporter assay. As shown in Fig. 2H, reduced luciferase activity was measured for wild‐type circFNDC3B in the presence of miR‐937‐5p mimics as compared with the mimics NC; however, its mutant exhibited no significant changes upon miR‐937‐5p mimics transfection. The direct binding between circFNDC3B and miR‐937‐5p was further confirmed by our RIP experiment (Fig. 2I) and we found that the increase of co‐precipitated circFNDC3B levels after being treated with miR‐937‐5p mimics was comparable to its mimics NC. In a word, circFNDC3B directly and negatively targets miR‐937‐5p in CRC.

### 3.3 Overexpression of circFNDC3B suppressed cell proliferation, migration and invasion in CRC, and the effects are compromised by miR‐937‐5p overexpression

**Fig. 3:**
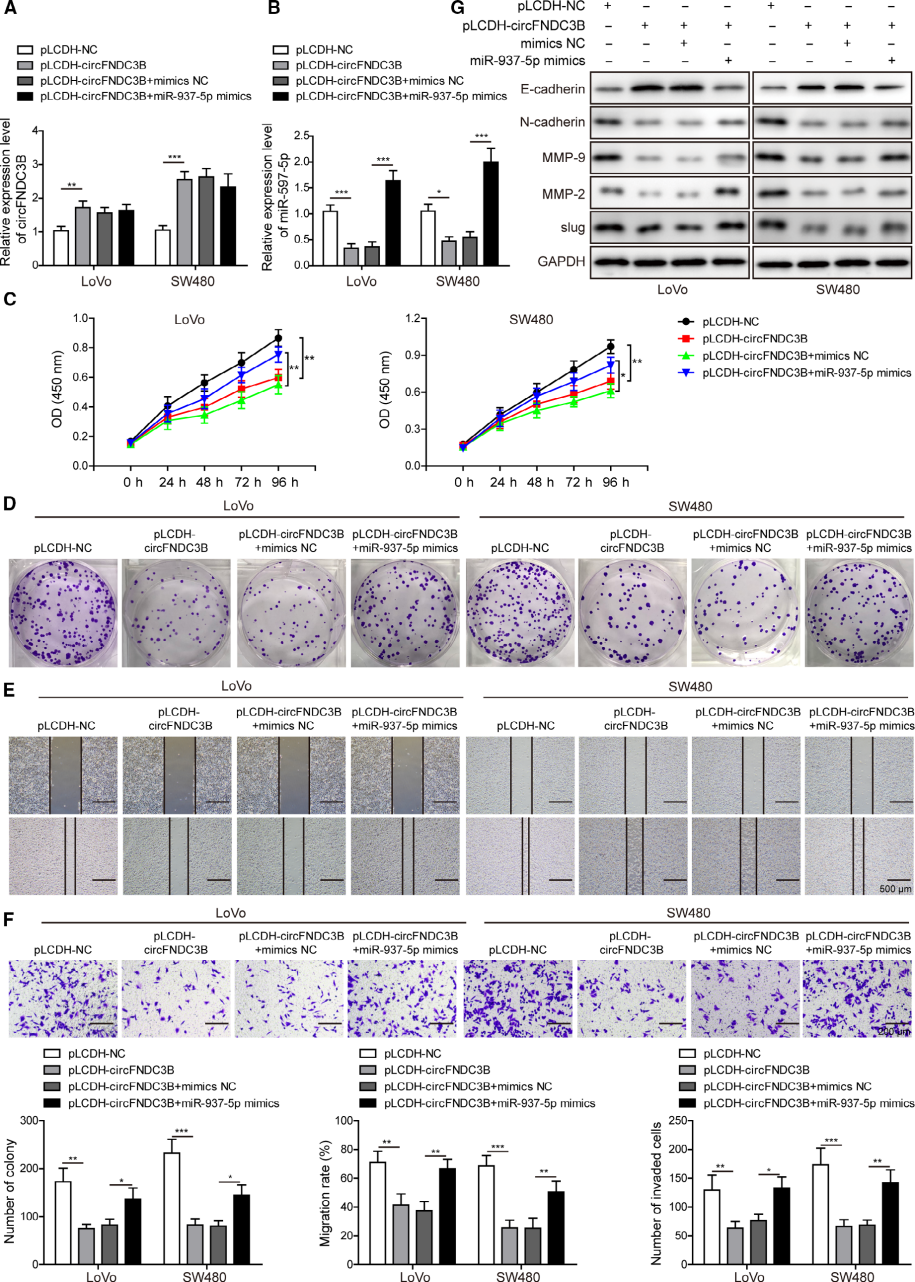
Overexpression of circFNDC3B suppressed cell proliferation, migration and invasion in CRC; the effects were compromised by miR‐937‐5p overexpression. (A) LoVo and SW480 cells were treated with pLCDH‐circFNDC3B alone or co‐transfected with miR‐937‐5p mimics, and the relative expression of circFNDC3B was compared by Q‐PCR. (B) Relative expression of miR‐937‐5p under indicated conditions was determined by Q‐PCR. (C) CCK‐8 assay was performed to determine the proliferation rate in the two cell lines. (D) Clone formation in the two cell lines was evaluated after the indicated treatments. (E) Migration ability of LoVo and SW480 cells in the specific groups was assessed by wound‐healing experiment. Scale bar: 500 μm. (F) Transwell assay was performed to measure the invasion ability in the two cell lines. Scale bar: 200 μm. (G) Differential expression levels of EMT regulators including E‐cadherin, N‐cadherin, MMP‐9, MMP‐2 and slug were determined by western blot. All the experiments were repeated three times. Statistical evaluation was performed using Student’s *t* test (two‐tailed) between two groups or one‐way ANOVA followed by Tukey’s *post hoc* test for multiple comparison. Data are expressed as mean ± SD. *P < 0.05; **P < 0.01; ***P < 0.001.

To investigate the effects of the dysregulated expression levels of circFNDC3B and miR‐937‐5p, we transfected LoVo and SW480 cells with lentiviruses carrying circFNDC3B alone or co‐transfected with miR‐937‐5p mimics. CircFNDC3B expression was largely increased upon virus infection and was not altered by co‐transfection of miR‐937‐5p mimics (Fig. 3A). However, circFNDC3B transfection effectively inhibited miR‐937‐5p expression, and the effect was suppressed by co‐transfection with miR‐937‐5p mimics (Fig. 3B). Cell proliferation and colony formation assays showed that LoVo and SW480 cells transfected with circFNDC3B exhibited the reduced cell viability (Fig. 3C) and colony formation ability (Fig. 3D) compared with the control group. Furthermore, circFNDC3B overexpression suppressed cell migration (Fig. 3E) and invasion (Fig. 3F) in both cell lines. Interestingly, these inhibitory effects of circFNDC3B on the CRC tumorigenic properties were partly abolished by the co‐transfection of miR‐937‐5p mimics.

Epithelial‐mesenchymal transition (EMT) is a process that is implicated in cell migration and invasion and that contributes essentially to the acquisition of metastatic potential in tumor cells. Therefore, we were prompted to test the potential effects of circFNDC3B and miR‐937‐5p on EMT induction during cancer progression. As observed in our western blot results, expression of the epithelial cell marker E‐cadherin was largely promoted by circFNDC3B; however, the mesenchymal cell marker N‐cadherin expression was eliminated, indicating circFNDC3B as a potent suppressor of EMT induction (Fig. 3G). In accordance with the results above, we observed a great reduction of the EMT‐inducing transcription factor slug as well as the EMT initiators MMP‐2 and MMP‐9 upon circFNDC3B overexpression, further substantiating the inhibitory function of circFNDC3B on EMT. In contrast, miR‐937‐5p overexpression promoted the EMT phenomenon and counteracted the effects of circFNDC3B. Collectively, our results show that circFNDC3B inhibit the tumorigenic and metastatic properties of CRC cells, which are conversely enhanced by miR‐937‐5p overexpression.

### 3.4 Overexpression of circFNDC3B suppressed CRC angiogenesis, which was reversed by miR‐937‐5p overexpression

**Fig. 4:**
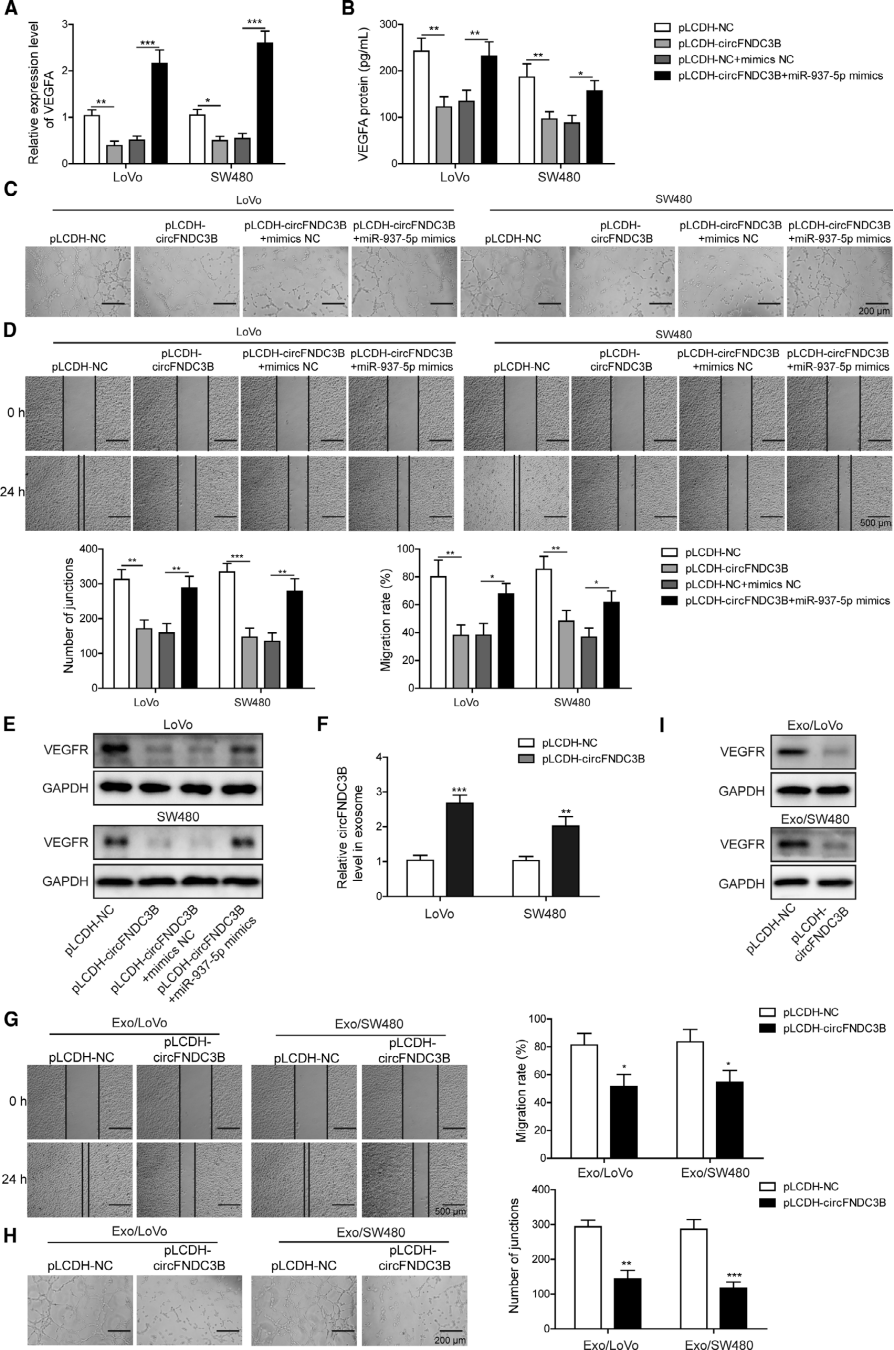
Overexpression of circFNDC3B suppressed CRC angiogenesis, which was reversed by miR‐937‐5p overexpression. (A, B) LoVo and SW480 cells were treated with pLCDH‐circFNDC3B alone or co‐transfected with miR‐937‐5p mimics, and the expression of VEGFA at mRNA and protein levels were determined by Q‐PCR (A) and ELISA (B), respectively. (C) HUVEC cells were treated with the CM collected from the cells above and the tube formation was observed under the microscope. Scale bar: 200 μm. (D) Changes of HUVEC migration abilities that were induced by the specific CM were assessed by wound‐healing experiment. Scale bar: 500 μm. (E) Western blot results showed that VEGFR expression in HUVEC was altered upon treatment of the indicated CM. (F) LoVo and SW480 cells were transfected with pLCDH‐NC or pLCDH‐circFNDC38, and the relative expression of circFNDC38 in exosomes was quantified by Q‐PCR. (G) Exosomes secreted by the cells in (F) were isolated and used to treat HUVEC. HUVEC migration was evaluated by a wound‐healing experiment upon treatment with the indicated exosomes. Scale bar: 500 μm. (H) Representative images of the tube formation in HUVEC upon treatment with the indicated exosomes are shown. Scale bar: 200 μm. (I) Western blot results showed that VEGFR expression in HUVEC was significantly decreased by treatment of the exosomes secreted by circFNDC3B overexpressing LoVo or SW480 cells. All the experiments were repeated three times. Statistical evaluation was performed using Student’s *t* test (two‐tailed) between two groups or one‐way ANOVA followed by Tukey’s *post hoc* test for multiple comparison. Data are expressed as mean ± SD. *P < 0.05; **P < 0.01; ***P < 0.001.

Cancer cell growth and tumor metastasis depend largely on angiogenesis, a process that is a critical factor in the extensive formation of new blood vessels during cancer progression [39]. The binding process of the angiogenic activator VEGFA produced by tumor cells to its receptor VEGFR on endothelial cells activates several nuclear signaling cascades and promotes endothelial cell proliferation. Aberrant VEGFA expression has been reported to be linked to poor clinical prognosis and metastasis in numerous cancer types. We detected a significant loss of VEGFA in both LoVo and SW480 cells after circFNDC3B transfection; however, overexpression of miR‐937‐5p restored VEGFA expression at both mRNA and protein levels (Fig. 4A,B). To explore further the molecular roles of circFNDC3B and miR‐937‐5p in angiogenesis, we incubated HUVEC with CM collected from circFNDC3B overexpressing CRC cells with or without miR‐937‐5p mimics transfection, and observed that tube formation (Fig. 4C) and cell migration (Fig. 4D) abilities, as well as VEGFR expression (Fig. 4E), of HUVEC were greatly inhibited after the incubation with circFNDC3B overexpressing cell culture CM. In contrast, the above effects were efficiently recovered by the treatment of miR‐937‐5p mimics (Fig. 4C–E). Next, we purified exosomes from the CM collected from circFNDC3B overexpressing cell culture. As hypothesized, we detected a significantly higher expression of circFNDC3B in exosomes isolated from CRC cells transfected with circFNDC3B vector (Fig. 4F). Furthermore, HUVEC incubated with purified circFNDC3B exosomes displayed the remarkably reduced tube formation and migration abilities (Fig. 4G,H) and VEGFR expression levels in HUVEC cells were almost eliminated after co‐culture with circFNDC3B exosomes (Fig. 4I). These findings suggest that circFNDC3B or circFNDC3B exosomes repress CRC angiogenesis by decreasing VEGFA/VEGFR expression levels, and that the inhibition effects are reversed by miR‐937‐5p overexpression.

### 3.5 MiR‐937‐5p directly target TIMP3 in CRC

**Fig. 5:**
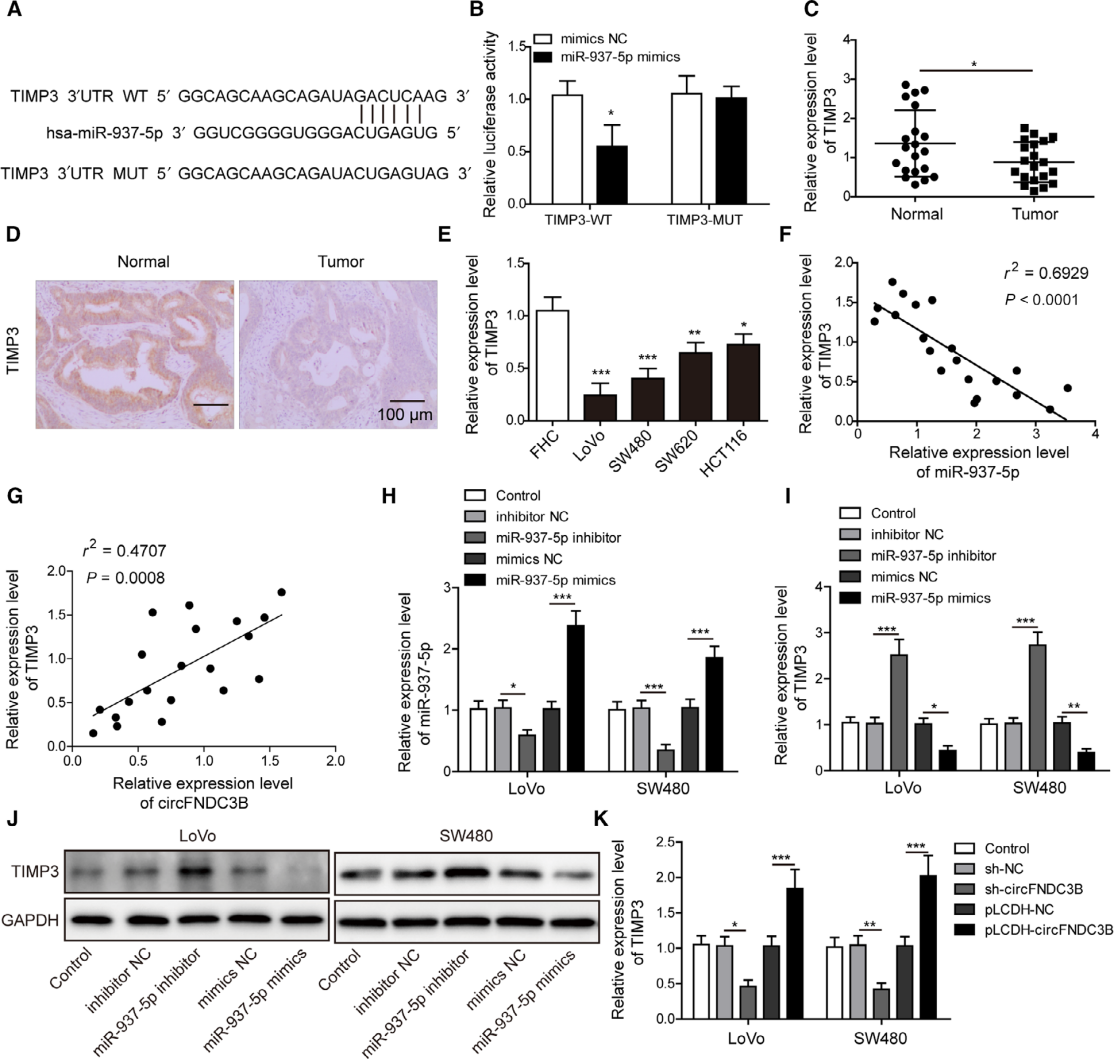
MiR‐937‐5p directly targeted TIMP3 in CRC. (A) The putative binding between miR‐937‐5p and TIMP3 was predicted and potential binding sites are shown. (B) 293T cells were co‐transfected with miR‐937‐5p mimics or mimics NC and TIMP3‐WT or TIMP3‐MUT luciferase reporter plasmids. Cells were harvested 48 h after transfection and relative luciferase activity was recorded. (C) Relative expression of TIMP3 was determined by Q‐PCR and compared between healthy individuals (*n* = 20) and CRC patients (*n* = 20). (D) Representative IHC images of TIMP3 in tissues from healthy individuals and CRC patients. Scale bar: 100 μm. (E) Q‐PCR was performed to compare the relative expression of TIMP3 in FHC and the other four CRC cell lines. (F) Correlation analysis between the expression of miR‐937‐5p and TIMP3 in CRC tissue samples. (G) Correlation analysis between the expression of circFNDC3B and TIMP3 in CRC tissue samples. (H, I) LoVo and SW480 cells were transfected with miR‐937‐5p inhibitor, miR‐937‐5p mimics or negative controls, and the relative expression levels of miR‐937‐5p (H) and TIMP3 (I) were measured by Q‐PCR. (J) Protein expression of TIMP3 under the conditions above was tested by western blot. (K) LoVo and SW480 cells were transfected with sh‐circFNDC3B, pLCDH‐circFNDC3B or their negative controls, and Q‐PCR was performed to determine the relative expression of TIMP3 mRNA. All the experiments were repeated three times. Statistical evaluation was performed using Student’s *t* test (two‐tailed) between two groups or one‐way ANOVA followed by Tukey’s *post hoc* test for multiple comparison. Data are expressed as mean ± SD. *P < 0.05; **P < 0.01; ***P < 0.001.

To identify the putative binding partner of miR‐937‐5p, we applied TargetScan tool and found TIMP3 to be a potential target for miR‐937‐5p (Fig. 5A). When we measured the decreased luciferase activity for TIMP3‐WT in the presence of miR‐937‐5p mimics as compared with the negative control, the luciferase activity was not changed for the TIMP3‐MUT, further confirming the direct binding of miR‐937‐5p and TIMP3 (Fig. 5B). Next, we compared the mRNA expression levels of TIMP3 in CRC tissues and in paired normal samples by Q‐PCR. In accordance with a previous report by Wang *et al*. [27], we observed that TIMP3 was markedly downregulated in CRC tissue samples (Fig. 5C). TIMP3 downregulation was further confirmed by immunohistochemistry (IHC) test of CRC tissues (Fig. 5D) and Q‐PCR analysis in CRC cell lines (Fig. 5E). Intriguingly, we found a significant negative correlation between expression levels of TIMP3 and miR‐937‐5p in CRC tissues (Fig. 5F), whereas the expression levels of circFNDC3B and TIMP3 were positively correlated with each other (Fig. 5G). To elucidate the regulatory interaction between TIMP3 and miR‐937‐5p, we infected LoVo and SW480 cells with lentiviruses carrying miR‐937‐5p mimics or inhibitor for stable expression or knockdown of miR‐937‐5p (Fig. 5H). Notably, TIMP3 expression was largely enhanced by the knockdown of miR‐937‐5p and eliminated by miR‐937‐5p overexpression at both mRNA (Fig. 5I) and protein (Fig. 5J) levels. In contrast, circFNDC3B overexpression enhanced TIMP3 expression, whereas it was inhibited by circFNDC3B knockdown (Fig. 5K). These results illustrate that miR‐937‐5p directly targets TIMP3 and the expression of TIMP3 is downregulated by miR‐937‐5p in CRC.

### 3.6 Knockdown of miR‐937‐5p suppresses cell proliferation, migration and invasion, which are reversed by TIMP3 knockdown

**Fig. 6:**
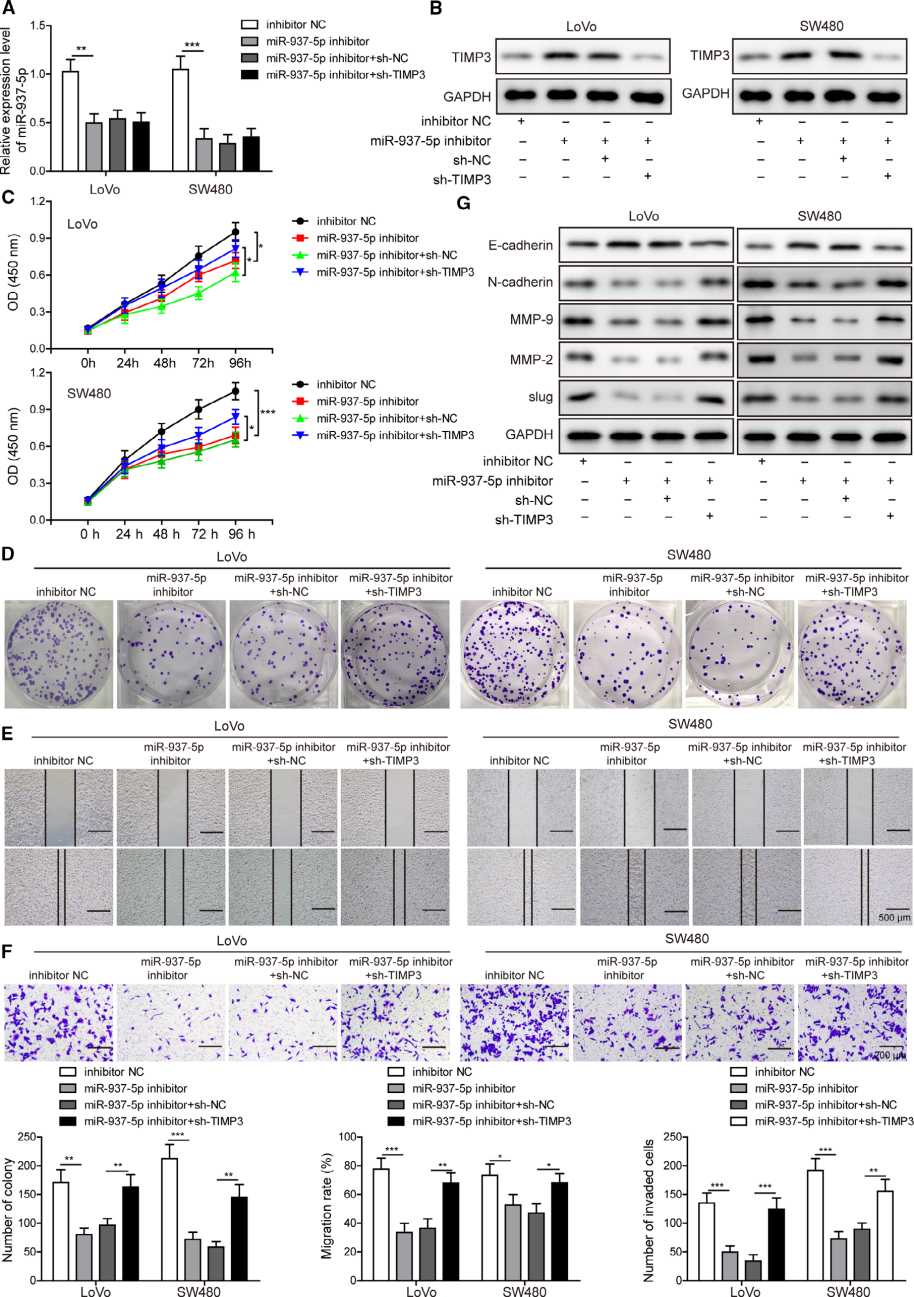
Knockdown of miR‐937‐5p suppressed cell proliferation, migration and invasion, which were reversed by TIMP3 knockdown. (A, B) LoVo and SW480 cells were transfected with miR‐937‐5p inhibitor and/or sh‐NC, sh‐TIMP3, and the expression levels of miR‐937‐5p as well as TIMP3 were measured by Q‐PCR (A) or western blot (B), respectively. (C) Cell proliferation rate in both cell lines under the specific conditions was determined by CCK‐8 assay. (D) Clone formation upon the indicated treatments was compared. (E) Migration rates of the cells above were evaluated by a wound‐healing experiment. Scale bar: 500 μm. (F) Invasive capacity of LoVo and SW480 cells was assessed by Transwell invasion assay. Scale bar: 200 μm. (G) Western blot was performed to measure the expression changes of EMT markers induced by miR‐937‐5p inhibitor and/or TIMP3 knockdown. All the experiments were repeated three times. Statistical evaluation was performed using Student’s *t* test (two‐tailed) between two groups or one‐way ANOVA followed by Tukey’s *post hoc* test for multiple comparison. Data are expressed as mean ± SD. *P < 0.05; **P < 0.01; ***P < 0.001.

To test the hypothesis whether dysregulated expression levels of miR‐937‐5p and TIMP3 in CRC were associated with tumorigenic and metastatic abilities, we transfected LoVo and SW480 cells with miR‐937‐5p inhibitor alone or co‐transfected with sh‐TIMP3 (Fig. 6A). Western blot analysis showed that TIMP3 expression was increased by miR‐937‐5p knockdown and inhibited by sh‐TIMP3 treatment (Fig. 6B). Furthermore, LoVo and SW480 cells transfected with miR‐937‐5p inhibitor showed reduced cell viability (Fig. 6C) and clone formation ability (Fig. 6D) compared with the control group. Similarly, migration assay (Fig. 6E) and invasion assay (Fig. 6F) showed that metastatic properties of CRC cells were suppressed by silencing of miR‐937‐5p expression. Interestingly, these inhibitory effects of miR‐937‐5p knockdown on the CRC tumorigenic and metastatic potential were partly abolished by the co‐transfection of sh‐TIMP3. Western blot analysis for the expression of EMT markers was carried out to test whether miR‐937‐5p and TIMP3 were implicated in the EMT process. MiR‐937‐5p inhibitor enhanced E‐cadherin expression and decreased the expression levels of N‐cadherin, slug, MMP‐2 and MMP‐9 (Fig. 6G), suggesting that EMT was inhibited by miR‐937‐5p knockdown. In contrast, TIMP3 knockdown exerted opposite effects and promoted the EMT process. Our results indicate knockdown of miR‐937‐5p suppresses cell proliferation, migration and invasion by inducing TIMP3.

### 3.7 Knockdown of miR‐937‐5p suppresses angiogenesis, which is reversed by TIMP3 knockdown

**Fig. 7:**
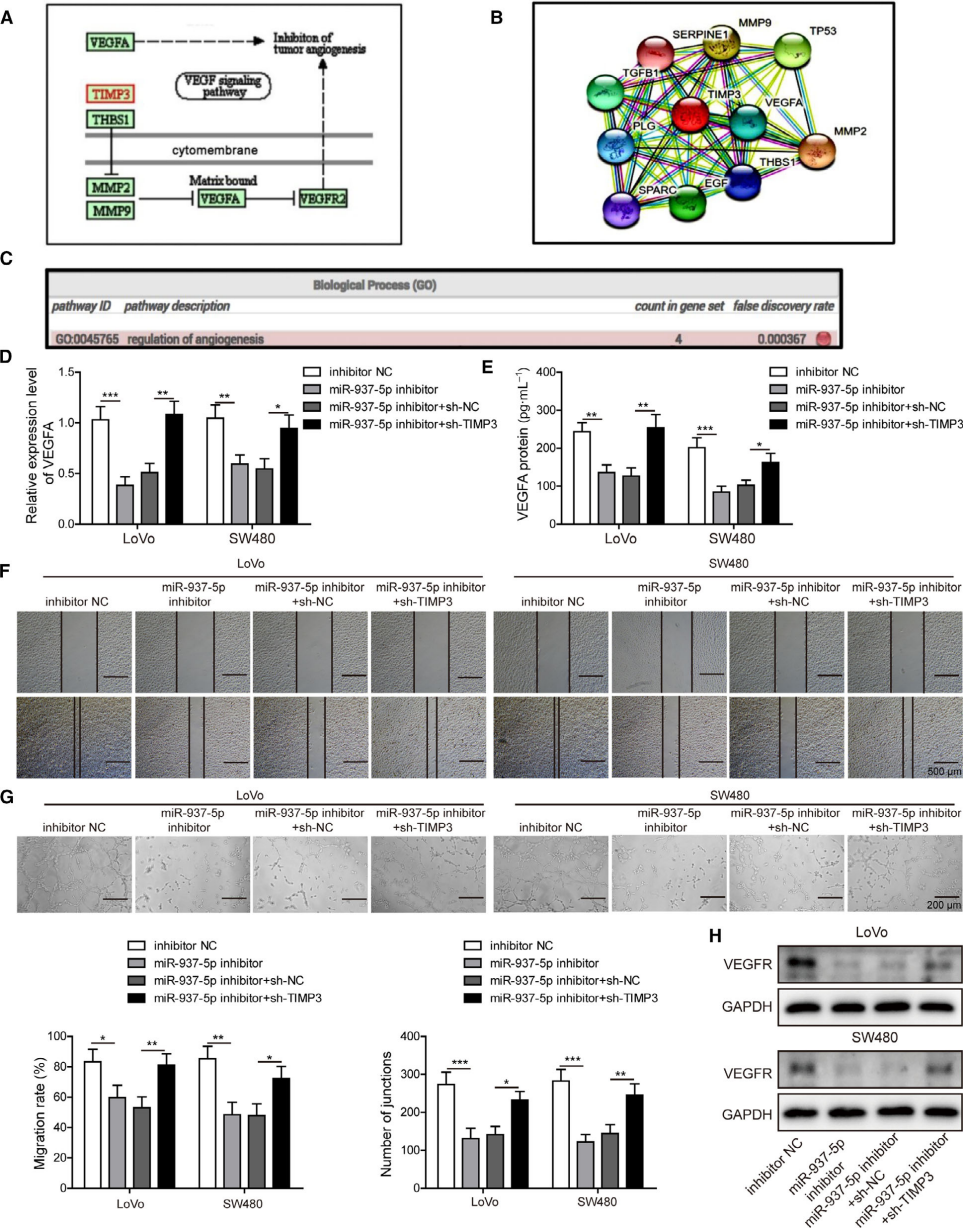
Knockdown of miR‐937‐5p suppressed angiogenesis, which was reversed by TIMP3 knockdown. (A) KEGG pathway database was accessed and TIMP3 was predicted to regulate angiogenesis by targeting the VEGFA/VEGFR signaling pathway. (B) The interaction between TIMP3 and VEGFA was revealed by STRING protein‐protein association network analysis. (C) GO analysis of the genes within the protein association network confirmed that the interaction between TIMP3 and VEGFA participated in the angiogenesis process. (D, E) LoVo and SW480 cells were transfected with miR‐937‐5p inhibitor, and/or sh‐NC, sh‐TIMP3, and the molecular expression of VEGFA was measured by Q‐PCR (D) and ELISA (E). (F) HUVEC was treated with the CM harvested from the cells above and changes of HUVEV migration capacity were evaluated with a wound‐healing experiment. Scale bar: 500 μm. (G) Representative images of the tube formation were shown by treating HUVEC using the indicated CM. Scale bar: 200 μm. (H) Western blot was performed to assess VEGFR expression in HUVEC treated with the CM. All the experiments were repeated three times. Statistical evaluation was performed using Student’s *t* test (two‐tailed) between two groups or one‐way ANOVA followed by Tukey’s *post hoc* test for multiple comparison. Data are expressed as mean ± SD. *P < 0.05; **P < 0.01; ***P < 0.001.

According to KEGG pathway database, STRING protein‐protein association network analysis and GO enrichment analysis, we predicted the interaction between TIMP3 and VEGFA and assumed that TIMP3 inhibited CRC angiogenesis by targeting the VEGFA/VEGFR pathway (Fig. 7A–C). Our hypothesis was supported by the observation that VEGFA expression was inhibited by miR‐937‐5p knockdown and largely enhanced by sh‐TIMP3 treatment at both mRNA (Fig. 7D) and protein (Fig. 7E) levels. Next, we incubated HUVEC with CM collected from CRC cells transfected with miR‐937‐5p inhibitor or sh‐TIMP3, and observed that cell migration (Fig. 7F) and tube formation (Fig. 7G), as well as VEGFR expression (Fig. 7H) in HUVEC cells, were inhibited by incubating the cells with the CM transfected with miR‐937‐5p inhibitor, and largely restored upon incubation with CM co‐transfected with sh‐TIMP3 (Fig. 7F–H). The results illustrate that miR‐937‐5p knockdown significantly suppresses the *in vitro* angiogenesis by directly targeting TIMP3.

### 3.8 CircFNDC3B‐induced phenotypes of CRC cells are reversed by TIMP3 knockdown

**Fig. 8:**
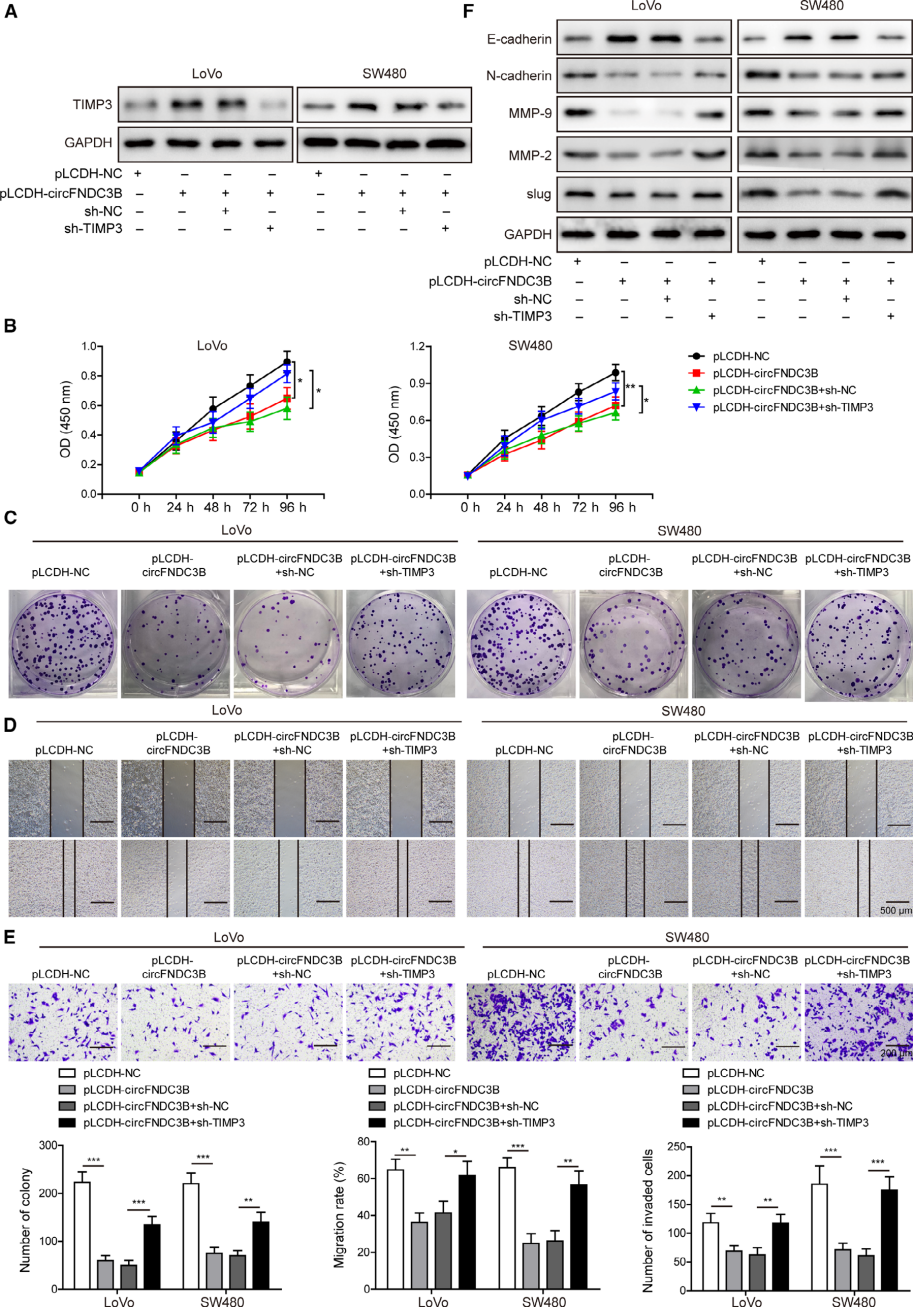
Overexpression of circFNDC3B suppressed cell proliferation, migration and invasion in CRC, and the effects were compromised by TIMP3 knockdown. (A) LoVo and SW480 cells were transfected with pLCDH‐circFNDC3B and/or sh‐NC, sh‐TIMP3, and protein expression of TIMP3 was determined by western blot. (B) Cell proliferation rate in both cell lines under the specific conditions was determined by CCK‐8 assay. (C) Clone formation upon the indicated treatments was compared. (D) Migration rates of the cells above were evaluated by a wound‐healing experiment. Scale bar: 500 μm. (E) Invasive capacity of LoVo and SW480 cells was assessed by Transwell invasion assay. Scale bar: 200 μm. (F) Western blot was performed to measure the expression changes of EMT markers induced by circFNDC3B overexpression and/or TIMP3 knockdown. All the experiments were repeated three times. Statistical evaluation was performed using Student’s *t* test (two‐tailed) between two groups or one‐way ANOVA followed by Tukey’s *post hoc* test for multiple comparison. Data are expressed as mean ± SD. *P < 0.05; **P < 0.01; ***P < 0.001.

To address the question whether circFNDC3B‐induced phenotypes were affected by the expression of TIMP3, LoVo and SW480 cells were transfected with pLCDH‐circFNDC3B alone or co‐transfected with sh‐TIMP3 vector (Fig. 8A). CCK‐8 assay showed that inhibition of TIMP3 expression largely promoted the cell viability which was suppressed by circFNDC3B overexpression (Fig. 8B). Accordingly, the reduced colony formation ability induced by circFNDC3B overexpression was significantly restored by TIMP3 knockdown in both cell lines (Fig. 8C). Furthermore, cells co‐transfected with sh‐TIMP3 regained migration (Fig. 8D) and invasion (Fig. 8E) abilities as compared with those transfected with circFNDC3B alone. Also, the inhibited EMT process caused by circFNDC3B overexpression was restored by TIMP3 knockdown (Fig. 8F).

**Fig. 9:**
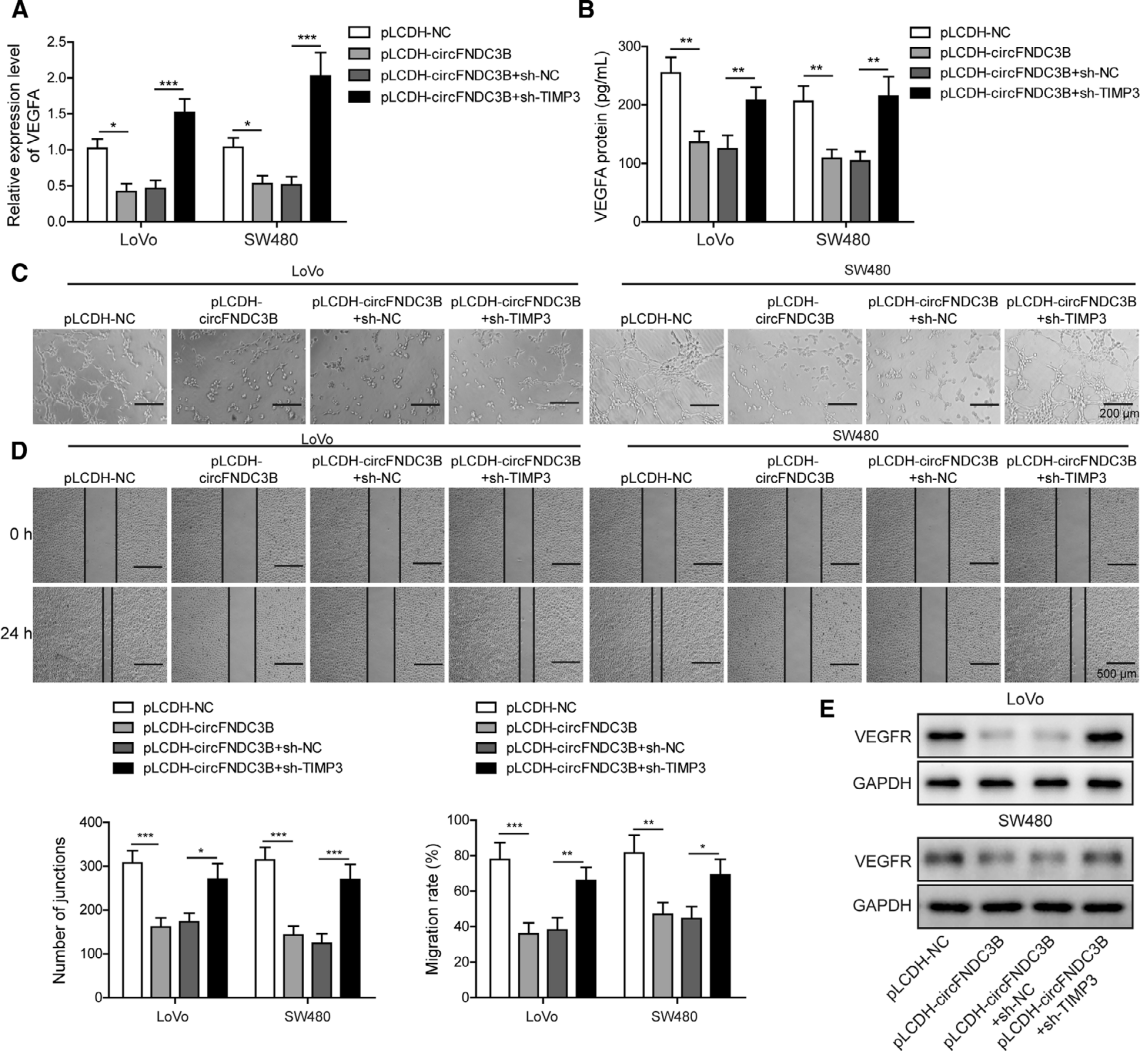
Overexpression of circFNDC3B suppressed CRC angiogenesis, which was reversed by TIMP3 knockdown. (A, B) LoVo and SW480 cells were transfected with pLCDH‐circFNDC3B and/or sh‐NC, sh‐TIMP3, and the expression levels of VEGFA were measured by Q‐PCR (A) or western blot (B) respectively. (C) HUVEC cells were treated with the CM collected from the cells above and the tube formation was observed under the microscope. Scale bar: 200 μm. (D) Changes of HUVEC migration abilities which were induced by the specific CM were assessed by a wound‐healing experiment. Scale bar: 500 μm. (E) Western blot results showed that VEGFR expression in HUVEC was altered upon treatment of the indicated CM. All the experiments were repeated three times. Statistical evaluation was performed using Student’s *t* test (two‐tailed) between two groups or one‐way ANOVA followed by Tukey’s *post hoc* test for multiple comparison. Data are expressed as mean ± SD. *P < 0.05; **P < 0.01; ***P < 0.001.

Next, we tested whether inhibition of angiogenic properties induced by circFNDC3B in CRC cells were altered by TIMP3 knockdown, and observed that upon sh‐TIMP3 co‐transfection, the expression of VEGFA, which was reduced by circFNDC3B overexpression, was markedly restored at both mRNA (Fig. 9A) and protein (Fig. 9B) levels. What is more, the decreased tube formation (Fig. 9C) and migration (Fig. 9D) abilities resulting from circFNDC3B overexpression were significantly restored by TIMP3 knockdown in both cell lines. In accord, VEGFR protein expression was clearly enhanced in cells co‐transfected with sh‐TIMP3 as compared with those transfected with circFNDC3B alone (Fig. 9E). Taken together, our results demonstrate that circFNDC3B‐induced tumorigenic and angiogenic phenotypes of CRC cells are abrogated by silencing of TIMP3.

### 3.9 *In vivo* overexpression of circFNDC3B or circFNDC3B‐exosome treatment suppressed CRC tumor growth, angiogenesis and liver metastasis

**Fig. 10:**
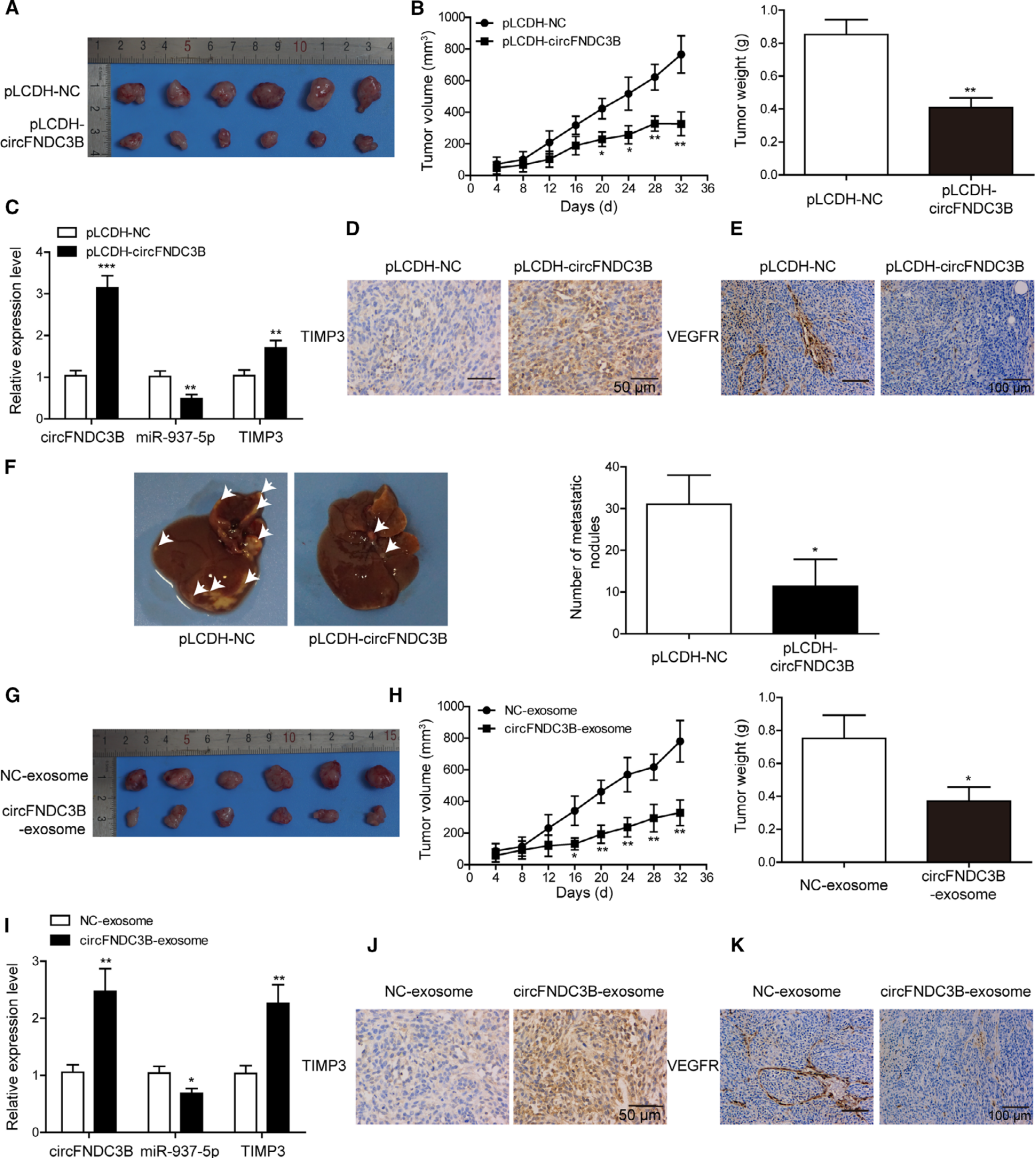
*In vivo* overexpression of circFNDC3B or circFNDC3B‐exosome treatment suppressed CRC tumor growth, angiogenesis and liver metastasis. (A) The CRC mice model that was injected with pLCDH‐circFNDC3B cells developed significantly smaller tumors than did the negative control. (B) Statistical analysis of the tumor volume and weight as measured in (A). (C) Relative expression levels of circFNDC3B, miR‐937‐5p and TIMP3 were compared in CRC tumors that were injected with pLCDH‐circFNDC3B or pLCDH‐NC. (D, E) IHC analysis showed the staining of TIMP3 (D, Scale bar: 50 μm) and VEFGR (E, Scale bar: 100 μm). (F) A liver metastasis model was established by tail vein injection of pLCDH‐circFNDC3B cells, and the number of metastatic nodules in liver was compared. (G) CRC mice model that was injected with pLCDH‐circFNDC3B exosomes developed significantly smaller tumors compared with those injected with pLCDH‐NC exosomes. (H) Statistical analysis of the tumor volume and weight as measured in (G). (I) Relative expression levels of circFNDC3B, miR‐937‐5p and TIMP3 were compared in CRC mice model that were injected with pLCDH‐circFNDC3B exosomes or pLCDH‐NC exosomes. (J, K) IHC analysis of the tumors from the mice above showed staining of TIMP3 (J, Scale bar: 50 μm) and VEFGR (K, Scale bar: 100 μm). All the experiments were repeated three times. Statistical evaluation was performed using Student’s *t* test (two‐tailed) between two groups or one‐way ANOVA followed by Tukey’s *post hoc* test for multiple comparison. Data are expressed as mean ± SD. *P < 0.05; **P < 0.01; ***P < 0.001.

To determine the *in vivo* function of circFNDC3B, we established a CRC animal model by injecting mice with LoVo cells that were transfected with lentiviruses encoding or not encoding circFNDC3B. We found that tumor volumes and weights were markedly reduced in mice injected with circFNDC3B overexpressing LoVo cells compared with control mice (Fig. 10A,B). Q‐PCR analysis of tumor tissues revealed that relative expression levels of circFNDC3B and TIMP3 were upregulated in circFNDC3B overexpressing mice, whereas miR‐937‐5p was downregulated (Fig. 10C). High protein expression of TIMP3 in circFNDC3B overexpressing mice was detected by IHC (Fig. 10D). Moreover, loss of VEGFR expression as visualized in IHC analysis suggested that angiogenesis was severely inhibited in circFNDC3B overexpressing mice compared with the control group (Fig. 10E). Liver is the most common metastatic site for CRC patients and liver metastasis is often associated with very low 5‐year survival rate [40]. We next generated liver metastasis model by injecting mice with LoVo cells transfected with circFNDC3B lentiviruses *via* tail vein; results showed that the number of metastatic nodules in liver was decreased by circFNDC3B (Fig. 10F).

Furthermore, we tested the circFNDC3B‐exosome effects on tumor growth and angiogenesis. Tumor volumes and weights (Fig. 10G,H) were decreased, respectively, in mice treated with circFNDC3B exosomes and mice treated with NC exosomes. Expression profiles of circFNDC3B, TIMP3 and miR‐937‐5p in tumor tissues were similar to results in Fig. 8C, whereby the first two were upregulated and the last downregulated (Fig. 10I). High expression of TIMP3 was also confirmed by IHC (Fig. 10J). In addition, the large loss of VEGFR expression indicated that angiogenesis was impaired by circFNDC3B exosomes (Fig. 10K). All these results prove that circFNDC3B or circFNDC3B‐exosome treatment suppresses CRC tumor growth, angiogenesis and liver metastasis *in vivo*.

### 3.10 *In vivo* knockdown of miR‐937‐5p suppresses tumor growth, angiogenesis and liver metastasis

**Fig. 11:**
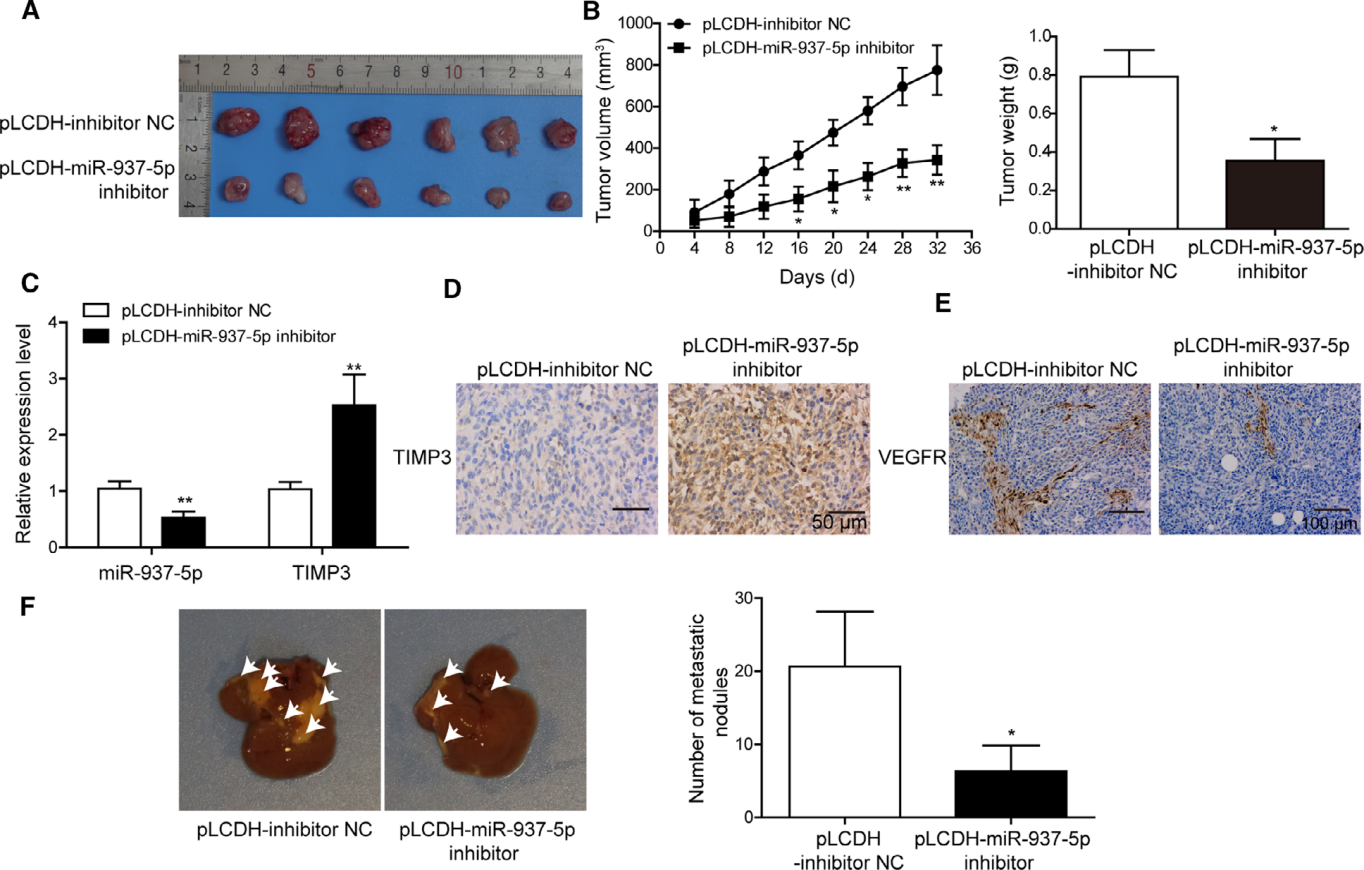
*In vivo* knockdown of miR‐937‐5p suppressed tumor growth, angiogenesis and liver metastasis. (A) A CRC mice model that was injected with pLCDH‐miR‐937‐5p inhibitor cells developed significantly smaller tumors compared with negative control. (B) Statistical analysis of the tumor volume and weight as measured in (A). (C) Relative expression levels of miR‐937‐5p and TIMP3 were compared in tumors injected with pLCDH‐miR‐937‐5p inhibitor or pLCDH‐inhibitor NC. (D, E) IHC analysis showed staining of TIMP3 (D, Scale bar: 50 μm) and VEFGR (E, Scale bar: 100 μm). (F) Liver metastasis model was established by tail vein injection of cells containing pLCDH‐miR‐937‐5p inhibitor or pLCDH‐inhibitor NC, and the number of metastatic nodules in liver was compared. All the experiments were repeated three times. Statistical evaluation was performed using Student’s *t* test (two‐tailed) between two groups or one‐way ANOVA followed by Tukey’s *post hoc* test for multiple comparison. Data are expressed as mean ± SD. *P < 0.05; **P < 0.01.

To examine the *in vivo* role of miR‐937‐5p in CRC, we generated a CRC animal model by injecting mice with LoVo cells that were transfected with lentiviruses carrying miR‐937‐5p inhibitor or not carrying the inhibitor. Tumor volumes and weights were significantly smaller in mice receiving miR‐937‐5p inhibitor than in the control group (Fig. 11A,B). Q‐PCR showed that miR‐937‐5p expression was reduced in mice receiving miR‐937‐5p inhibitor, but TIMP3 expression was higher than in the inhibitor NC group (Fig. 11C). IHC staining further confirmed the high expression of TIMP3 after knockdown of miR‐937‐5p (Fig. 11D). Further, we detected reduced expression of VEGFR, suggesting impaired angiogenesis in mice with miR‐937‐5p knockdown (Fig. 11E). Liver metastasis was also effectively suppressed by miR‐937‐5p inhibition (Fig. 11F). These findings strongly indicate that silencing of miR‐937‐5p could suppress CRC tumor growth, angiogenesis and liver metastasis *in vivo*.

## 4 Discussion

Colorectal cancer is one of the most deadly cancer forms with relatively low survival rate and only 35% of patients survive 5 years after diagnosis [3, 41]. Current treatments for CRC usually involve surgery, chemotherapy or radiation and have led to a profound improvement in life expectancy during last several decades [42, 43]. However, due to the inherent complexities of CRC arising from the genetic heterogeneity, unclear relationship between clinical prognosis and TNM stages and relatively high risks of metastasis and recurrence [5], treatment of CRC patients remains a major challenge, especially in those with distant metastases. In the exploratory study, we found that expression levels of circFNDC3B, miR‐937‐5p and TIMP3 were dysregulated in CRC cell lines, tissue samples or exosomes in plasma samples. Moreover, we have demonstrated for the first time that CRC tumor growth, angiogenesis and liver metastasis are governed by circFNDC3B/miR‐937‐5p/TIMP3 axis through direct binding and elucidated that circFNDC3B or circFNDC3B exosomes induce TIMP3 to suppress angiogenesis to inhibit CRC progression by targeting miR‐937‐5p.

Compelling evidence in the last decade indicated that circRNA play important roles in various cancer types [44, 45, 46, 47, 48]. In the present study, we compared the circRNA expression profiles in plasma exosomes isolated from CRC patients and healthy controls. By conducting GO enrichment analysis on the 387 circRNA which were significantly differentially expressed, we identified eight that were potentially associated with cancer metastasis, whereas the expression of circFNDC3B was most dominantly downregulated in exosomes of CRC cells. Moreover, we detected lower expression of circFNDC3B in CRC cell lines than in FHC from normal colonic mucosa; circFNDC3B expression level in CRC tissues also showed downregulation, further supporting our discovery.

Previously, circFNDC3B was reported to play tumor‐suppressive or oncogenic roles by direct binding to different miRNA molecules [9, 11, 12]. However, the regulatory axis underlying the significantly dysregulated expression of circFNDC3B in CRC remains unexplored. Here we identified specific binding sites between circFNDC3B and miR‐937‐5p, and expression levels of circFNDC3B and miR‐937‐5p were negatively correlated with each other in CRC cell lines and tissue samples. On the other hand, we found that tumorigenic and metastatic properties shown by proliferation, migration and invasion in CRC were suppressed by circFNDC3B overexpression. Interestingly, angiogenesis proved by tube formation and VEGFR expression in HUVEC was also inhibited by circFNDC3B overexpression or circFNDC3B‐enriched exosomes, further confirming the tumor‐suppressive function of circFNDC3B in CRC progression by inhibition of the angiogenesis process. In contrast, the tumor inhibitory effects were counteracted by miR‐937‐5p overexpression. Our results for the first time have unraveled the regulatory mechanisms between circFNDC3B and miR‐937‐5p and described the tumor‐suppressive roles of circFNDC3B‐ or circFNDC3B‐enriched exosomes in CRC. However, how circFNDC3B‐enriched exosomes regulate tumor angiogenesis and which molecules are involved in this process remain questions for further investigation.

We next identified TIMP3 as the downstream target of miR‐937‐5p in both CRC cells and tissue samples and discovered that miR‐937‐5p, which acted as an oncogene in CRC, inhibited TIMP3 activity by direct downregulation of its expression. We demonstrated in the present study that knockdown of miR‐937‐5p suppressed cell proliferation, migration, invasion and angiogenesis. TIMP3 is considered a tumor suppressor in different cancer types [27, 28, 29, 30, 31]. Further, we observed that the inhibitory functions of circFNDC3B on tumorigenic, metastatic and angiogenic properties of CRC cells were largely counteracted by TIMP3 knockdown. In accordance with the previous report by Qi *et al*. [32], we demonstrated that TIMP3 knockdown enhanced VEGFR expression and accelerated CRC tumor growth, migration and invasion as well as angiogenesis. Through the KEGG pathway database and STRING protein‐protein association network analysis, TIMP3 was also predicted to regulate angiogenesis by targeting the VEGFA/VEGFR signaling pathway. These results collectively suggest that TIMP3 is targeted by miR‐937‐5p in CRC and tightly regulates the tumorigenic and metastatic properties, as well as angiogenesis during CRC development.

Consistent with our *in vitro* experimental results, we observed that tumor growth and angiogenesis in CRC mice model were effectively inhibited by circFNDC3B overexpression, circFNDC3B‐enriched exosomes or miR‐937‐5p knockdown. Among the diverse places that CRC cells can spread to, liver is the most common site for metastasis [4] and liver metastasis is often associated with poor prognosis [49]. If not treated, CRC patients with liver metastasis can only survive for up to 20 months. Our *in vivo* analysis showed for the first time that liver metastasis was efficiently suppressed by circFNDC3B overexpression or miR‐937‐5p knockdown.

## 5 Conclusion

In summary, we report in this study that circFNDC3B/miR‐937‐5p/TIMP3 axis governs CRC tumor progression, angiogenesis and liver metastasis and demonstrate that circFNDC3B induces TIMP3 to suppress angiogenesis to inhibit CRC progression by targeting miR‐937‐5p. We also demonstrate that circFNDC3B exosomes repressed CRC angiogenesis by decreasing VEGFR expression. Our study provides a novel CRC pathogenesis and a basis for available therapy modules to develop new anti‐CRC treatments.

6

## Author contributions

WZ and J‐FZ are guarantors of the integrity of the entire study and provided the definition of intellectual content. WZ provided study concepts. J‐FZ designed the study. WZ and W‐TL were involved in literature research. YL was involved in clinical studies and acquired data. YL and W‐TL were involved in experimental studies and statistical analysis. YL and YL analyzed the data. WZ and J‐FZ prepared the manuscript. J‐FZ edited and reviewed the manuscript.

## Data Availability

The datasets used and/or analyzed during the current study are available from the corresponding author on reasonable request.
